# Occurrence and Risk Factors of Dog Bites in Northern Indigenous Communities: A Scoping Review

**DOI:** 10.3389/fvets.2022.777640

**Published:** 2022-04-18

**Authors:** Laurence Daigle, Léa Delesalle, André Ravel, Barrie Ford, Cécile Aenishaenslin

**Affiliations:** ^1^Département de Pathologie et Microbiologie, Faculté de Médecine Vétérinaire, Université de Montréal, Saint-Hyacinthe, QC, Canada; ^2^Groupe de Recherche en Épidémiologie des Zoonoses et Santé Publique (GREZOSP), Faculté de Médecine Vétérinaire, Université de Montréal, Saint-Hyacinthe, QC, Canada; ^3^Centre de Recherche en Santé Publique de l'Université de Montréal et du CIUSSS du Centre-Sud-de-l'île-de-Montréal, Montréal, QC, Canada; ^4^Makivik Corporation, Kuujjuaq, QC, Canada

**Keywords:** scoping review, dog bites, epidemiology, Indigenous, northern communities

## Abstract

The relationship between northern Indigenous people and dogs has evolved over the past years alongside events such as colonization, settlement, proliferation of snowmobiling and other socio-cultural and environmental changes. These changes have had negative impacts on this relationship, and with the endemic presence of arctic fox rabies, dog bites have become an important public health burden. The objective of this study was to synthesize the state of knowledge regarding the occurrence of dog bites and associated risk factors in the specific context of northern Indigenous communities. A scoping review was conducted in seven bibliographic databases, from June 2018 to May 2020. From this search, 257 original studies were identified and eight papers were included for final analysis. Annual occurrence of dog bites in northern Indigenous communities ranged from 0.61 to 59.6/10,000 inhabitants. Dog bites affected 27–62.9% of the population in those regions during their lifetime. Very few studies compared the occurrence of dog bites between people living in northern communities with other populations or settings, but available evidence suggests that Indigenous people living in northern communities are at higher risk of dog bites than the rest of the population. Several individual and environmental risk factors were identified in the selected studies, although the strength of evidence varied significantly. Age (children) and gender (male) were well documented individual risk factors. Other factors, such as organizational barriers to dog management and lack of access to veterinary services, were identified and discussed by several authors. The results of this study support concerns about the higher risk of bites in northern Indigenous communities, and underscore the urgent need for more research into the contextual and environmental factors that impact the mitigation of these risks.

## Introduction

About 7 million people are currently living in the Arctic, about 10% of whom are Indigenous (1). This statistic varies widely from one territory to another and depends on the definition of “Arctic” and “Indigenous.” For example, the proportion of Indigenous people exceeds 75% in Greenland and northeastern Canada (2, 3). Northern Indigenous people are organized across more than 40 nations (4). Saami, Nenets, and Evenk people in Eurasia, and Inuit, Cree and Innu people in the Americas are among the main Indigenous people living in the arctic and subarctic territories. Many northern Indigenous communities have relied on dogs to hunt, travel or protect their belongings and families (5). However, this ancient partnership has also been profoundly affected by colonization, settlement, proliferation of snowmobiling and other socio-cultural and environmental changes (5, 6). Despite these recent (and sometime rapid) changes, dogs still play an important role in many northern Indigenous communities. In North American communities, dogs are often abundant and roam free, and the lack of access to veterinary services, such as neutering, makes it difficult to control the canine population (7).

Bites are a common dog-related human health threat, and their incidence depends greatly on the context. In Canada, 0 to 9 dog bites per 10,000 inhabitants (median: 1.9) were reported by 22 municipalities in a study occurring between 2003 and 2005 (8). Dog bites can lead to physical injury, impact mental health (post-traumatic stress) and can affect well-being, by creating concerns about safety and conflicts and misunderstandings (e.g., whether or not to tie up the dogs, take better care of dogs…) between community members (9). In northern and/or Indigenous communities of North America, dog bites are a common cause of medical consultation, even though their true incidence is difficult to assess due to various factors, including the lack of mandatory reporting at the national and regional scales (10, 11).

Dog bites are also the transmission route of various zoonotic diseases, such as secondary bacterial infections with *Pasteurella* sp., *Streptococcus* sp., *Staphylococcus* sp. and other anaerobic agents and viral diseases, such as rabies virus (12, 13). The latter still kills ~59,000 people annually worldwide (14). The Arctic Rabies Virus Variant (ARVV) is endemic among arctic fox populations in many regions of the Arctic, including northern Canada, Alaska, Greenland, Svalbard and northern Russia (15–18). In Canada, 105 positive animal rabies cases were detected in 2021, of which 6 were dogs (6/105) (19). The annual incidence of rabies in foxes in Nunavik (northern Quebec, Canada) is 1.6 cases/year (15). Consequently, exposure of humans to ARVV through dog bites happens regularly in these areas (11, 20). In some regions, such as Nunavik, there are initiatives to vaccinate dogs against rabies, but these initiatives are not mandatory and are often limited (21, 22). Limited access to dog rabies vaccination in some communities may increase the risk of transmission, and sometimes forces the administration of post-exposure prophylaxis as the only way to prevent human infection after a bite. Knowing that climate change may increase the incidence of ARVV in wild and domestic animals in the next decades by modifying the movements and interactions of arctic and red fox populations, public health concerns are increasing with regard to the risk of bites in these particular regions (15, 23).

Effective prevention of dog bites and rabies exposure requires a good understanding of their risk factors. In general, dog breed or size and reproductive status have been suggested as possible dog-related risk factors. Other human-related factors which have been proved or suspected by several authors include age (children), gender (male) and behavior of the victim as well as the interactions between these factors (20, 24–26). Previous studies on dog bite risk factors were conducted in diverse contexts (geographic locations, urban and rural, among others). Given the particular context of northern Indigenous communities, understanding the specificity and importance of those risk factors is crucial.

This scoping review aims to assess the current state of knowledge on the occurrence of dog bites and their risk factors in the specific context of northern Indigenous communities. More precisely, the research questions behind this scoping review are as follows:

(1) What is known about the occurrence of dog bites in the specific context of northern Indigenous communities and how it compares to other contexts (such as southern, urban or non-Indigenous contexts)?
(2) What is known about dog bite risk factors specific to northern Indigenous communities?

This review is being conducted as preliminary work for a study aiming to improve the understanding of dog bite risks factors in Northern Indigenous communities in the province of Quebec, Canada. In Quebec, “northern communities” mainly refers to communities of Nunavik, a region which extends to the 55th parallel. Furthermore, this location is also known to have animals carrying Arctic fox rabies almost every year (7, 11, 18).

## Methods

### Protocol and Registration

We conducted an exploratory scoping review applying the guidance developed by Peters et al. (27) from the Joanna Briggs Institute and five Joanna Briggs Collaborating Centers, and following the PRISMA-ScR checklist developed by Peters et al. (27) and Tricco et al. (28). This type of scoping review was selected with the objectives of mapping the body of literature on the subject and identifying research gaps relative to dog bites in northern communities. The main steps are detailed in this section: (1) identifying the research question, (2) identifying relevant studies following a plan that includes the databases, the terms used and the other criteria, (3) selecting the studies with specific inclusion and exclusion criteria, (4) charting the data, and (5) collating, summarizing and reporting results to provide an overview of the literature on the subject. The protocol was registered with the Open Science Framework on June 5, 2020 (29).

### Eligibility Criteria

#### Inclusion Criteria

The studies included in this scoping review needed to have relevant and sufficient content related to dog bites in northern and Indigenous communities. Northern communities in this review are defined as all the communities located north of the 55th parallel. As explain above, the 55th parallel is also the southern limit of Nunavik, Quebec, Canada, and the region where rabies is considered endemic in arctic fox populations; this scoping review is prior to a study on dog bites in this region. Since there is no real consensus on a definition for Indigenous peoples due to the variability of these communities, the term “Indigenous community” in this review is defined by the characteristics stated by the United Nations Permanent Forum on Indigenous Issues: (1) self-identification as Indigenous peoples at the individual level and accepted by the community as their member, (2) historical continuity with pre-colonial and/or pre-settler societies, (3) strong link to territories and surrounding natural resources, (4) distinct social, economic or political systems, (5) distinct language, culture and beliefs, (6) form non-dominant groups of society, and (7) resolve to maintain and reproduce their ancestral environments and systems as distinct peoples and communities (30). All types of studies were considered (original studies, reviews) but opinion papers were excluded.

Additional inclusion criteria were to be written in English or French and to be peer-reviewed. All articles published and referenced up to May 2020 were included.

#### Exclusion Criteria

Papers were excluded if the content was focused on other non-Indigenous groups of people or if the content addressed poorly the occurrence of dog bites or the risk factors related to dog bites, e.g., other animal bites, other diseases related to dogs or treatments after injuries caused by bites. Finally, if the full text was not retrievable, the paper was removed from the selection.

### Information Sources

We used four health electronic databases [CAB abstract, Medline (MEDLINE, RRID:SCR_002185), Global Health and Native Health] and three social sciences electronic databases (Autochtonia, Popline, and Sociological abstract). References lists were also examined to extract additional relevant articles.

### Search

The research strategy has been developed with the collaboration of the librarian at the Faculté de Médecine Vétérinaire, Université de Montréal. The search was conducted in two steps (phases 1 and 2). An initial search occurred in July and August 2018, in electronic databases (phase 1). Then, a final search was done in May 2020 to find new relevant papers (phase 2). We used combinations of key words related to the themes “dog bites,” “northern community” and “Indigenous community,” as “dog bites”, “dog aggression,” “northern,” “Nordic,” “Nunavik,” “arctic,” “subarctic,” “autochthonous,” “Indigenous,” “american indian,” “native,” and “first nation” (see Supplementary Table 1 in the Appendix for complete equations used).

### Selection of Sources of Evidence

All the records found in databases and additional resources, as the references lists, were imported in a data-collecting software, Zotero 5.0 (Zotero, RRID:SCR_013784), and exported in a Microsoft Excel 2017 spreadsheet (Microsoft Excel, RRID:SCR_016137) in order to proceed with the classification. One reviewer (LDa) conducted the first selection of studies based on their titles and abstracts and removed: (1) duplicates, (2) studies in a language other than English and French, and (3) studies which were not peer reviewed. The reasons of exclusions were noted in the spreadsheets. Two reviewers (LDa and LDe) carried out the last screening step where the remaining articles were read in full. The criteria for the inclusion and the exclusion of articles are listed above. Three reviewers (LDa, CA, and AR) approved the studies included for the further analysis.

### Data Charting Process

A grid, in a single Microsoft Excel 2017 spreadsheet (Microsoft Excel, RRID:SCR_016137), was made by two reviewers (LDa and LDe) to extract information from articles. The characteristics of each study included were extracted by two reviewers (LDa and LDe) independently and compiled in the grid. The results were discussed by the reviewers and a consensus on the information extracted was made.

### Data Item

We summarized the data from the articles according to the characteristics listed in Box 1.

Box 1

**Extraction fields used for the scoping review.**
(1) Authors
(2) Year of publication
(3) Title
(4) Journal
(5) Publication type
(6) Inclusion phase
(7) Aims/Purpose
(8) Source origin/Place
(9) Study period
(10) Study design
(11) Study population, sample size
(12) Occurrence of dog bites and method of measurement
(13) Risk/Protective factors influencing dog bites
(14) Importance accorded to the topic of interest
(15) Comparison with other contexts
(16) Authors' limits and perspectives

### Critical Appraisal of Individual Sources of Evidence

For all the papers included, the outcomes (occurrence of dog bites) were interpreted considering the measurement method and the p-value (if applicable). Each risk factor influencing dog bites was classified according to whether an association was demonstrated statistically in the article or whether it was only stated hypothetically. The studies were also classified by the importance accorded to the main objectives of the scoping review. For example, when the purpose of the paper was about dog bites and the title included “dog bites,” the importance given was then major. When the subject of dog bites was not the main topic but was covered in some part(s) of the article or when the protocol included means for investigating the bites, the importance given was secondary.

### Synthesis of Results

The synthesis of results is presented as follows: (1) distribution of articles over time, (2) distribution of the articles among the different Indigenous communities, (3) occurrence of dog bites according to the study design, the definition of dog bites, the study population and sample size (if applicable), (4) studied risk factors, and (5) importance of the subject of dog bites among the articles. Identified risk factors were classified using the One Health framework, which advocates considering the interdependence between the health and well-being of humans, animals and the environment to gain a better understanding of the global complexity (31, 32). We extracted the study design and methods used to estimate the occurrence according to their epidemiological evidence, whether with a qualitative or a quantitative design. In the quantitative design, the factors were identified either with or without a statistically significant association with the risk of a bite or a suggestion of a possible association. Finally, we present a qualitative synthesis of the review results.

## Results

### Selection of Sources of Evidence

**Figure 1:**
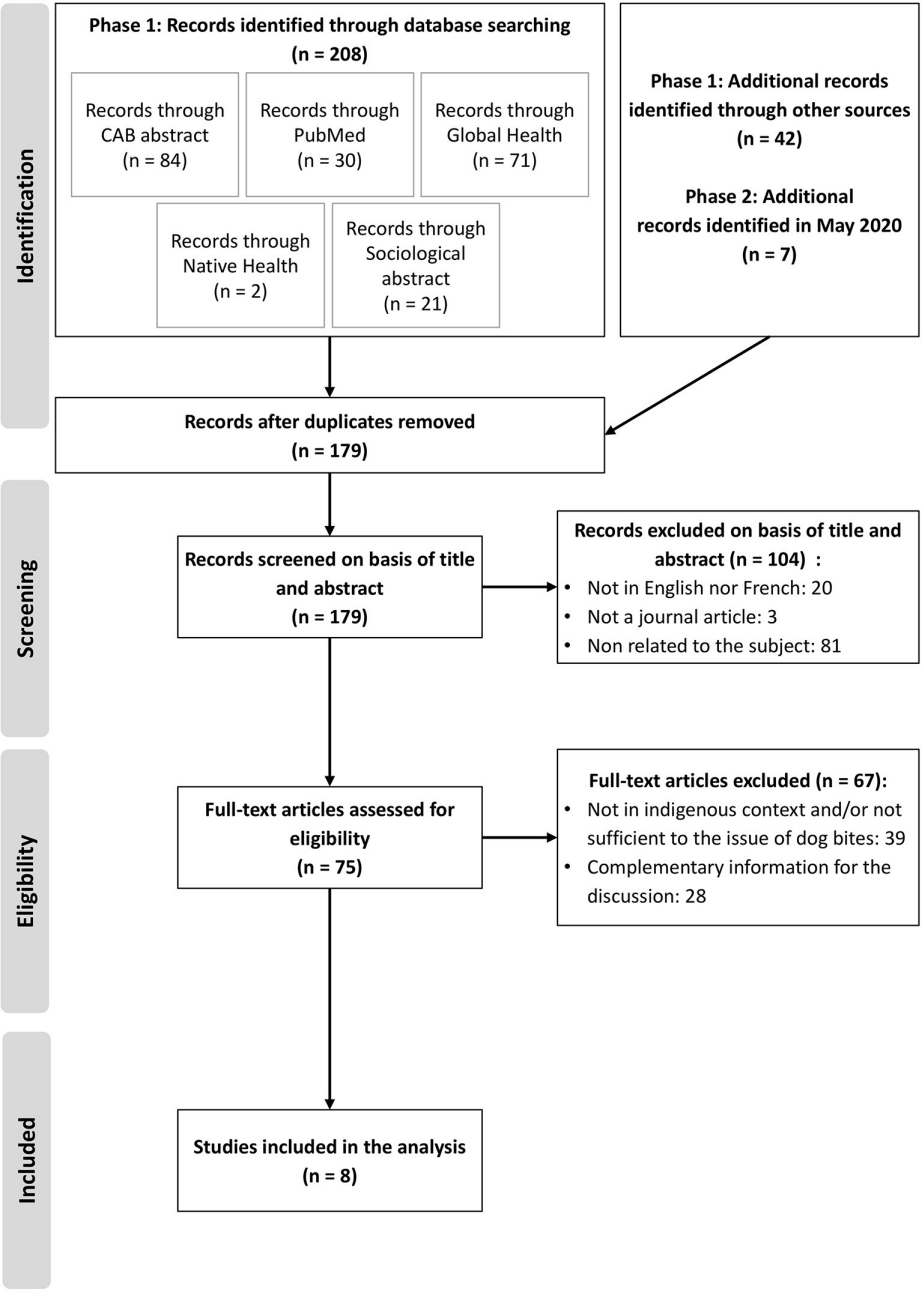
PRISMA flowchart presenting the selection process (33).

From the database search, 257 records were identified in two phases, from June 2018 to May 2020 (Figure 1). A total of 75 full-text articles were assessed for the relevance and the importance of the northern Indigenous context and the dog bite issue. In total, 8 papers were included for final analysis, seven papers from phase 1 (7, 10, 20, 34–37) and one from phase 2 (11).

### Characteristics of Sources of Evidence

The first article included was published in 2007 (35), but most studies (6/8) were published between 2010 and 2019 (Table 1). The northern Indigenous communities included or mentioned were Inuit from Nunavik, Canada (3/8) (7, 11, 20), Sahtu from Northwest Territories, Canada (1/8) (34), Cree and Assiniboine from Saskatchewan, Canada (2/8) (36, 37), and unspecified Natives from Alaska, USA (2/8) (10, 35). One of the United States studies (10) also compared dog bite injuries among children from non-Nordic (American Indian) and Nordic (Alaska Native) Indigenous communities and mentioned the Navajo and other American Indian communities from the USA as well. We found no publications from Eurasia.

**Table 1:** General characteristics of included scoping reviews (*n* = 8) (total percentages may exceed 100% as publications have been classified in more than one category).

**Characteristic**	***n* (%)**
**Publication year**	
2000–2009	1 (12.5%)
2010–2019	6 (75%)
2020	1 (12.5%)
**Indigenous communities**	
Inuit from Nunavik, Canada	3 (37.5%)
Sahtu from Northwest Territories, Canada	1 (12.5%)
Alaska Natives from Alaska, USA	2 (25%)
Cree and Assiniboine from Saskatchewan, Canada	2 (25%)
**Study design**	
Quantitative (Based on health data*)*	3 (37.5%)
Qualitative	1 (12.5%)
Mixed	4 (50%)
**Importance accorded to the main subject**	
Major	3 (37.5%)
Secondary	5 (62.5%)

The studies used different methodologies, including quantitative, qualitative, and mixed-method designs (Table 1). In less than half of the studies (3/8), the importance accorded to dog bites was major and it was secondary in all the other studies (5/8).

### Sources of Dog Bite Data

Two main data sources were used to estimate dog bite occurrence: (1) dog bites as reported by local, regional or national health authorities (based on health records) and (2) dog bites as reported by study participants (self-reported bites) (see Table 2).

**Table 2:** Dog bite occurrence in northern Indigenous communities.

**References**	**Study population**	**Period**	**Data sources**	**Sample size**	Case definition^*^	**Occurrence of dog bites**
Aenishaenslin et al. (7)	Kuujjuaq, Nunavik, Canada	Fall 2015	Questionnaires	*n* = 35 Inuit and 32 non-Inuit	Dog's owners and family member bitten or scratched in their lifetime	Inuit: 62.9% (22/35) Non-Inuit: 15.6% (5/32)
Aenishaenslin et al. (20)	Nunavik inhabitants, Canada	1996–2009	Health records	*n* = 112 consultations for potential exposure to a rabid animal	Consultations for a potential rabies exposure (bites or scratches)	*n* = 76 dog bites
Bjork et al. (10)	Alaskan native children (<20 years old), USA	2001–2008	Health records	26 hospitalizations 2,530 outpatient visits	Hospitalizations and outpatient visits with diagnosis of dog bite	H: 6.1/100,000 per year O: 596.4/100,000 per year
Brook et al. (34)	Sahtu of NWT, Canada	2008–2009	Questionnaires	*n* = 67 student respondents *n* = 41 dog owners	Students and dog owners bitten at least once in their life	42% students (*N* = 67) 27% dog owners (*N* = 41)
Castrodale (35)	Alaskan native, USA	1991–2002	Health records	Alaska population in 1997: 609,655 peoples 288 dog-related cases in total in Alaska for the period	Hospitalizations ≥ 1 day with a diagnosis of dog bites	9.3/100,000 per year
Mediouni et al. (11)	Nunavik inhabitants, Canada	2008–2017	Health records	Nunavik population in 2017: 13,549*^a^* 293 dog-related cases	Consultations for a potential rabies exposure	2.5/1,000 per year (0.45 to 4.6/1,000) 293 dog-related cases
Schurer et al. (36)	Cree and Nakota of SK, Canada	2006–2013	Health records	4 out of 6 communities of the health area included 53 dogs attacks out of 59 animal attacks *(total population of the area in 2016: 1,155^b^)*	Consultations following an animal bite and/or scratch	3/1,000 attacks per year (95% CL: 1.0–6.6/1,000) 90% from dogs (53 of 59 attacks)

* *The case definition refers to the considered items that are related to dog bites or altercations in order to include the case as data*.

a *Population of RCM of Administration régionale Kativik (38)*.

b *Data from Statistic Canada (39)*.

Among the seven included studies that estimated dog bite occurrence, five used health records. Two of those five extracted data from health records (10, 35). Two estimated the incidence of potential rabies exposure, therefore included dog bites and bites by other species (e.g., foxes), scratches or other at-risk contacts (11, 20).

The two studies that used self-reported bites conducted cross-sectional surveys with Indigenous people from two northern communities (7, 34). They assessed the prevalence of dog-related injuries by asking participants if they had been bitten or scratched at least once in their life.

### Dog Bite Occurrence

According to the studies based on health records, annual occurrence in northern Indigenous communities ranges from 0.61 to 59.6/10,000 inhabitants, whereas surveys suggest that dog bites could affect 27–62.9% of the population in those regions during their lifetime (Table 2). Some studies (3/8) compared dog bite occurrence between northern Indigenous population and others, and only two of those have statistically tested the difference (Table 3).

**Table 3:** Comparison of dog bite occurrence between northern Indigenous populations and others.

**References**	**Study site**	**Nordic Indigenous**	**Other populations*^a^***	**Test**
Aenishaenslin et al. (7)	Kuujjuaq, QC, Canada	Inuit: 62.9% (22/35)	Non-Inuit: 15.6% (5/32)	Pearson's χ^2^ *p* < 0.001
Bjork et al. (10)	Alaska, USA	Hospitalizations: 6.1/100,000	Other USA natives: 3.4/100,000 Global USA: 3.9/100,000	*None*
		Outpatients: 596.4/100,000	Other USA natives: 392.4/100,000	*None*
Castrodale (35)	Alaska	Natives: 9.3/100,000	Non-natives: 2.8/100,000	*None*
		Hospitalizations: 40.4% were Indigenous people	% of natives in Alaska population: 20%	χ^2^ = 114, *p* < 0.0001
		Mean duration*^b^*: 4.6 days [95%CI = 3.7–5.5]	2.5 days [95%CI = 2.2–2.9]	p < 0.0001

a *The term “Other populations” refers to a population that is either non-Indigenous, non-Nordic, or both*.

b *Mean duration represents the period of hospitalization (in days)*.

### Risk Factors Associated With Dog Bites

The information about the different risk factors explored in each study is synthesized in Table 4. In general, risk factors were more often discussed than assessed in the included studies. Some human-related factors associated with higher risk of bites were identified both by quantitative and qualitative methods, including age (children), gender (male), behavior toward dogs, and Indigenous status. Dog related factors (breed, size, roles, gender, ownership and numbers of dogs implicated) were sometimes briefly discussed but their effect was never quantified. Structural and environmental factors were often considered (never quantified), especially in studies using a qualitative design. Lack of veterinary services, geographic remoteness, lack of legislation, density of dogs (overpopulation), free roaming, and seasonality (summer) were all factors reported to increase the occurrence of dog bites in these settings.

**Table 4:** Dog bite risk factors identified by the studies included in the scoping review (some studies may have been classified in more than one categories).

	**Study design**			
**Risk factors**	**Qualitative design**	**Quantitative design**		**Reported as hypothesis or cited from literature**
	**Evidence of importance by qualitative methods**	**Statistically significant association with the occurrence of bites**	**Suggesting a possible association without having proven it**	
**Individual human factors**				
Age (children)	(11)	0	(10, 11, 34–36)	(7)
Gender (male)	0	(10), [11^*^]	(11, 34, 35)	(36)
Behavior toward dog (conflictual/provoked)	(7, 11, 37)	0	(11, 34, 35), [36^*^]	0
Sociocultural characteristics (ethnicity)	(36)	(35)	(7, 10, 11)	0
**Dog factors**				
Breeds/size	0	0	(35)	0
Function/role	(11)	0	(35)	0
Gender and reproductive status	0	0	0	(36)
Ownership or presence of a keeper	0	0	(35)	(37)
Number (lone dogs)	0	0	0	0
**Structural and environmental factors**				
Lack of veterinary service or animal control resources	(11, 36)	0	(10)	(7, 11, 34, 37)
Geographic remoteness	0	0	0	(34, 36)
Lack of legislative interventions	(7, 11)	0	(10)	(7, 36, 37)
Density of dogs in the community (overpopulation)	0	0	(10)	(7, 11, 34)
Free roaming	(7, 11), [7, 37^*^]	0	[35^*^]	(7, 37)
Seasonality (temporal variations)	(11)	(11)	(36)	0

[*] *Contradictory result*.

Age was the most often identified risk factor in the included studies (6/8). Five papers found differences in the frequency of dog bites between these subgroups in their descriptive analysis (10, 11, 34–36). Brook et al. found that children under 17 years old represent up to 69% of the total victims (34).

Two studies (2/8) have shown an interaction between age and gender. In Bjork et al. the risk ratio (RR) of being bitten by a dog for male children was higher compared with female children (1.8; 95% CI, 1.3–2.6) for the Indigenous people (10). In Mediouni et al. boys and girls under 14 were equally represented in potential rabies exposures (mostly caused by dog bites), but men were overrepresented among victims over the age of 15 (11). For gender alone, males are generally overrepresented in four of the studies. The hypothesis raised for the overrepresentation of men in certain studies would be related to their riskier activities, such as hunting and mushing (11).

Inconsistent results were found in the different papers regarding behavior toward dogs (conflictual/provoked). Five studies report that dog bites may be most often provoked by the victims (7, 11, 34, 35, 37). Two studies (2/5) quantified this statement with 48% (34) and 56% (11) of dog bites that were provoked. However, Schurer et al. showed that 67% of the bites described were unprovoked (36). Furthermore, the presence of free-roaming dogs was sometimes suggested as a predisposing factor to dog bites. Some authors reported from interviews with community members or health professionals that loose dogs could increase the risk of dog aggression (7, 11). However, several studies mentioned that tying up dogs could make them less socialized, more aggressive and promote risky behavior, whereas free roaming dogs have some advantages, such as providing companionship and protection for the communities as a whole (7, 37). Indeed, Castrodale showed that many dog bites are caused by a dog tied up or in a closed space (35).

Several authors also discussed the protective factors against bites. These approaches and solutions were generally mentioned briefly in the included articles, even though we found no study that has tested those hypotheses. This included preventive education (4/8) (10, 20, 35, 37), access to veterinary services (5/8) (7, 11, 34, 36, 37), implication of para-veterinarians (2/8) (34, 37), collaborative approach in a One Health perspective (5/8) (7, 20, 34, 36, 37), law and legislation enforcement (2/8) (7, 34), density diminution and stabilization of the dog population (3/8) (20, 36, 37) and dog bite surveillance enhancements (1/8) (36).

## Discussion

This review is the first to compile available evidence on the occurrence of dog bites and their risk factors in northern Indigenous communities.

### Dog Bites Occurrence

Although this is a recent topic of interest in research and evidence is still scarce, our results suggest that Indigenous people living in northern communities are at higher risk of dog bites than the rest of the population. Indeed, in all studies that have compared the risk of dog bites in different contexts, regardless of the design and measurement method, the occurrence was higher in northern Indigenous people than in: (1) the general US or Canada population, (2) non-northern Indigenous people, (3) non-Indigenous inhabitants of northern communities, or (4) non-Indigenous inhabitants of other rural or remote areas. Around the world, Indigenous people are generally more prone to poorer health outcomes, such as higher rates of infectious diseases, mental disorders, nutritional deficiencies and infant mortalities (40). These findings on dog bites are concerning, and reveal an additional health inequity that affect Indigenous people. Addressing these issues is also relevant in term of the Sustainable Development Goals, in particular number 3 (“Ensure healthy lives and promote well-being for all at all ages”) and number 10 (“Reduce inequality within and among countries”) (41).

However, our review also undercover significant methodological gaps, which should be addressed in future studies. First, none of the studies used a longitudinal design, making it hard to robustly quantify the effect of potential risk factors at the individual level (e.g., age, gender, behaviors). Second, the demographic characteristics of the sample used to estimate dog bites occurrence was not always reported in the included studies, making it difficult to evaluate the representativeness of the sample.

Both self-reports and health records were used to estimate the occurrence of bites, leading to very different estimates. Health records are useful secondary sources of data but they may underestimate the occurrence since not everyone will seek medical services after a bite. Furthermore, a potential bias may arise for the proportions of the age groups affected. Indeed children, given their small size, are often bitten on the head, the neck and the face (11, 42, 43). Consequently, their injuries are more likely to require medical attention and thereby to be recorded by health authorities, causing a potential selection bias. In contrast, self-reported dog bites, collected using population surveys, may better reflect the true incidence of bites if a representative sample is achieved, since bites not reported to health authorities can be included. Surveys are subject to memory bias, even if the traumatic aspect of dog bites probably limits it. Often limited to adult participants, surveys should include questions relative to the bite history in the household to avoid underestimation of the burden of dog bites in children (7). We noticed differences in the case definition of a dog bite, which makes comparison between studies difficult. This is a methodological issue commonly noted by other authors (44). Also, for the studies reporting the incidence of potential rabies exposure (11, 20), the data sources did not always specify the species or route of exposure involved, therefore it is possible that they exclude a few data related to dog bites that were not recorded as such, leading to an underestimation of dog bites.

Surprisingly, included studies focused only on North American arctic and subarctic regions, overlooking northern Eurasia. Indeed, reindeer is the main subsistence resource for a lot of Eurasian Indigenous nations, even if dogs also play a role for transportation, herding and hunting (45–48). However, a few authors reported concerns among Saami and Nenets about an increasing number of stray dogs abandoned by shift workers, and of wolfdog breeding (48). There seems to be a lack of documentation on this subject and evidence is needed on the occurrence and risk factors of dog bites in northern Eurasia.

### Risk Factors

**Figure 2:**
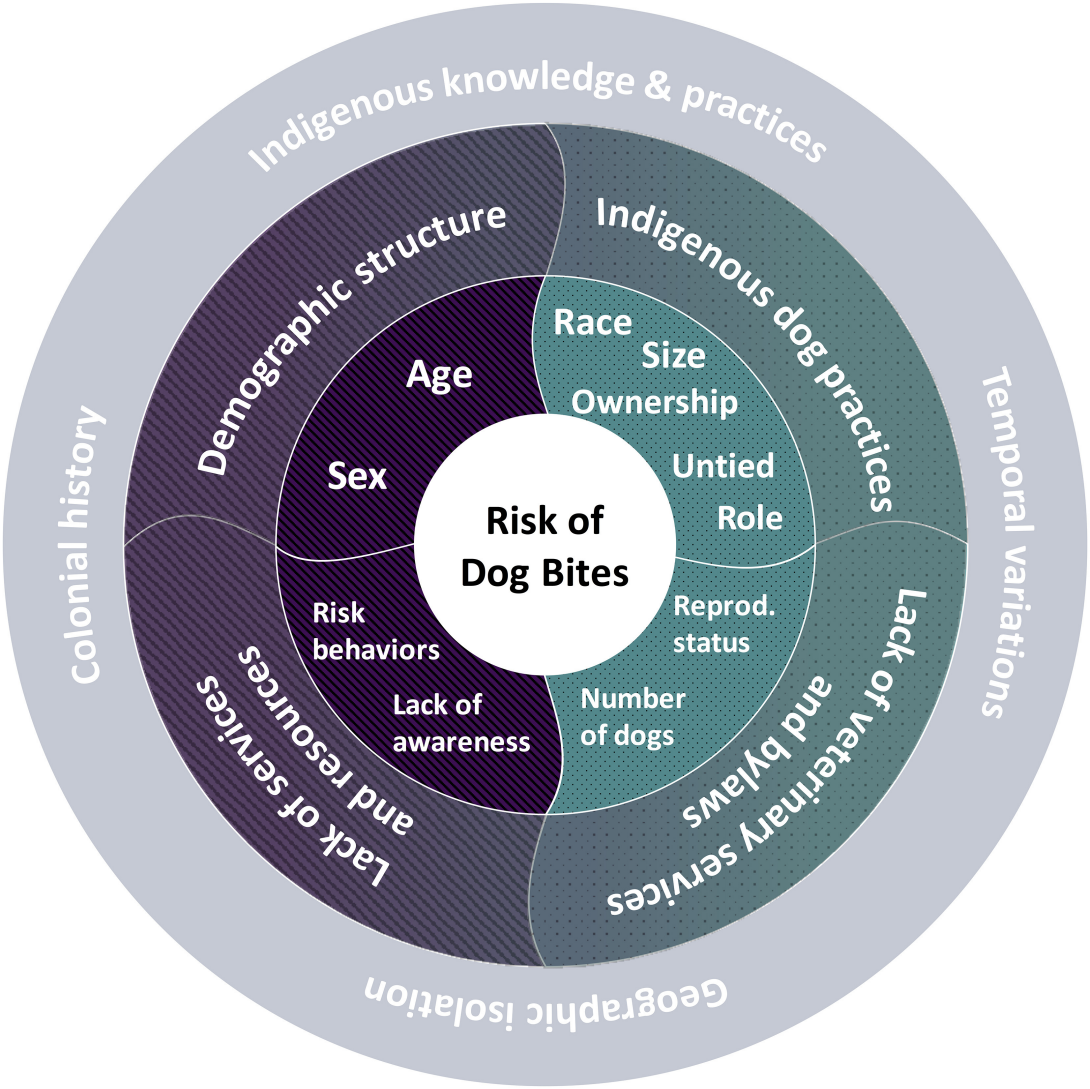
Factors capable of influencing the risk of dog bites in northern Indigenous communities, ranked from individual/familial factors (such as age, sex, dogs' ownership, and behavior toward dogs), to community factors (such as legislation and veterinary service), and contextual factors (such as seasonality and remoteness). (Hatched purple area), Human related factors; (Dotted turquoise area), Dog related factors; (Gray area), Contextual factors.

Some of the individual factors highlighted in this review are well-known to influence the occurrence of dog bites in other contexts. It is the case for age and gender, which tend to interact (24, 25, 49–51). Provocative behavior toward dogs, often by children, is also frequently mentioned as a risk factor in the literature (24, 52, 53). It is not specific to northern Indigenous communities (54, 55). Moreover, its effect on dog bites is unclear. On one hand, most of the included studies suggest that a bigger proportion of bites were “provoked” by the victim, which generally means that the dog had bitten defensively or out of fear, for example to defend its food, to protect its litter or in response to annoyance or mistreatment (44, 51). Those defensive and fear bites seem to happen preferentially if the dog is tied up, in a confined space or on its territory (house, yard). Risky behaviors of children have been targeted, amongst other factors, to highlight the importance of their consideration in future prevention programs (56). On the other hand, Schurer et al. assert that “unprovoked” bites were more frequent (36). Unprovoked bites can sometimes relate to predation. Predation bites are more likely to happen with packs of loose dogs, and generally cause more severe wounds (57, 58). The reviewed papers also discussed the role of factors more specifically related to Indigenous northern communities, including sociocultural characteristics, ethnicity and some structural and environmental factors. Figure 2 summarizes individual, community and contextual risk factors explored in the included papers.

One striking observation that comes from this synthesis is that several of those context-related factors, like Indigenous knowledge and practices or colonial history, appears difficult or impossible to change and are sometimes controversial (26, 54, 59, 60). The presence or density of free-roaming dogs is a good example. In this case, disagreement between authors could be linked to a lack of consensus on the definition of “free-roaming.” According to World Society for the Protection of Animals (2008), “roaming dogs are defined as dogs that are on public areas and not currently under direct control [and] this term is often used inter-changeably with ‘free roaming’, ‘free ranging,’ or ‘stray’ dogs” (61). The dog roaming loose could be either owned or unowned by someone, at different types of property. However, Jervis et al. criticize the western perception of free-roaming, which often considers this lack of canine restriction as pathological or a community dysfunction. They stipulate that there be an “ethnocentric bias that sees all roaming dogs as strays or feral, born of a construct of “pet ownership” whereby a dog can only belong to someone—and perhaps be loved—if it is restricted” (62). The concepts of “pet ownership” and domestication in relation to Indigenous communities are criticized by many other authors (5, 63). Cohen et al. reported that the dogs were not seen as property, but rather as part of the family (64). They then introduce the concept of “keepers” instead of “owners,” which invokes a vision where the dog can walk from house to house (multiple keepers) and can be loose. Different terms are therefore employed for approximately the same risk factors and several problems emerges in relation to definitions. This is a good illustration of the lack of appropriate language to study dog-related issues specific to Indigenous communities. Partnership with and validation of appropriate methods (e.g., terms used in a data collection instrument) by Indigenous communities appear as a crucial condition to advancing knowledge on this topic. Similarly, Dhillon et al. highlight that, despite the fact that several authors mention density of dogs and canine overpopulation as potential risk factors, none of them have established a clear causal link with the risk of dog bites (59).

As for occurrence, the capacity to explore risk factors in the studies depends on the data collection methods. Health records often provide standardized information on individual factors socio-demographic data about the victim (age, sex, village), and may also include data on the dog's breed and reproductive status. However, information is often lacking contextual variables. Indeed, in their studies on dog bite and rabies exposure in Nunavik (Quebec), Mediouni et al. reported a lot of missing data for exposure type, exposure site and use of PEP. In most cases, the commentary field was not filled, giving no information on the circumstances of the bite (11). Surveys, on the contrary, could allow a more in-depth exploration of the circumstances of bites, and document the knowledge, attitudes and behaviors of victims toward dogs.

Notably, several studies also used qualitative methods (e.g., individual interviews) imbedded in mixed-method study designs. Here, it allows a better understanding of contextual factors, which are difficult to highlight with a purely quantitative approach. Such factors may be more specific to northern Indigenous communities, like the lack of veterinary services and geographic isolation.

### Specificities of Northern Indigenous Communities

The reasons why northern Indigenous people appear more exposed to dog bites remain unclear. Indeed, most of the risk factors highlighted in this review aren't specific to this context (like age, gender or behavior toward dogs). Limited access to veterinary services and geographical isolation may be present in other remote areas. Canine overpopulation and high proportion of free-roaming dogs have been described in several African, South American and Asian communities (65–67). According to several authors, dog bites tend to be more frequent in low-income areas (68–70). Indigenous people tend to have lower income levels and higher poverty rates than non-Indigenous people (71, 72). Attributing health differences to ethnic or racial differences is always problematic, as it can foster inappropriate bias and lead to underestimation of the influence of social and health inequities. Indeed, in the context of dog bites, some risk factors, such as lower income and the lack of animal health services, are the consequences of years of colonial practices that have profoundly harmed Indigenous communities across the world. Understanding the social and cultural context is important to design effective and culturally adapted preventive interventions in Indigenous communities, but the role of ethnicity as a risk factor should be interpreted with caution (40, 73, 74). Importantly, we find no evidence of dog bites studies led or conducted by Indigenous researchers and/or communities, or which explicitly integrates Indigenous knowledge's in its research methods or interpretation. Since dogs have been part of the lifestyle of most northern Indigenous communities for centuries, addressing this gap would contribute to advancing knowledge and understanding of this complex issue.

### Limitations

This scoping review used a recognized and rigorous protocol through all steps of the methodology. A combination of search methods (databases from different disciplines, reference lists, internet search, snowball technique) was used to ensure the widest and the most accurate search possible. The literature was rescanned in May 2020 to include new articles that were yet published during the first phase. However, this scoping review may not have identified all the papers about dog bites in Nordic and Indigenous communities.

Key words used for this scoping review may also have unintentionally excluded publications concerning certain areas, as Indigenous people designation vary across countries. In addition, although our research included papers in English or French, our key words were only in English. Although unlikely, it may be possible that articles from Eurasia were unknowingly excluded in this way. Despite this limitation, a quick scan of the selected databases with key words specific to Eurasian Indigenous nations (for example Saami AND “dog bite,” or Evenk AND “dog bites,” etc.) tends to confirm a lack of international scientific literature about dog bites in Indigenous communities of northern Eurasia.

## Conclusion

This scoping review testifies to the significant knowledge gap concerning the occurrence of dog bites and their risk factors in northern Indigenous communities. The heterogeneity in data collection strategies, the absence of longitudinal designs and the lack of information on the representativeness of the sampled populations limits obtaining an accurate picture of the situation. More studies are needed to better understand the context particularities, and to allow the development of effective animal and public health interventions. Nevertheless, our review confirms that previously studied northern Indigenous people tend to be more exposed to dog bites than any other population in Canada or the US. Our findings also highlight the complexity of this health issue, which involves multiple factors, from humans and animals to social, structural and environmental; all likely to interact. Being systemic, multidisciplinary and intersectoral, the One Health approach could offer a framework fully addressing this complexity. Moreover, Indigenous knowledge and perspectives should be acknowledged, in order to better define local particularities and to facilitate the appropriation of the findings by the communities that need them.

## Author Contributions

LDa (45%) and LDe (35%) research and sorting of articles, analysis and interpretation of articles, and writing of the literature review. AR (7%) and CA (10%) contribution to the analysis and interpretation and revision of the manuscript. BF (3%) final revision of the manuscript. All authors contributed to the article and approved the submitted version.

## Funding

This study was provided by the *Fond du Centenaire* of Faculté de Médecine Vétérinaire at Université de Montréal, the *Fonds de recherche du Québec – Santé (FRQS)*, the Northern Scientific Training Program (NSTP), and the Canadian Institutes of Health Research (CIHR). The funders were not involved in the study design, collection, analysis, interpretation of data, the writing of this article or the decision to submit it for publication.

## Conflict of Interest

BF was employed by Makivik Corporation. The remaining authors declare that the research was conducted in the absence of any commercial or financial relationships that could be construed as a potential conflict of interest.

## Publisher's Note

All claims expressed in this article are solely those of the authors and do not necessarily represent those of their affiliated organizations, or those of the publisher, the editors and the reviewers. Any product that may be evaluated in this article, or claim that may be made by its manufacturer, is not guaranteed or endorsed by the publisher.
