# Erratum: Occurrence and Risk Factors of Dog Bites in Northern Indigenous Communities: A Scoping Review

**DOI:** 10.3389/fvets.2022.971946

**Published:** 2022-07-18

**Authors:** Frontiers Production Office

**Affiliations:** Frontiers Media SA, Lausanne, Switzerland

**Keywords:** scoping review, dog bites, epidemiology, indigenous, northern communities

Due to a production error, the references were incorrectly numbered in Table 4 and in one paragraph.

A correction has been made to the section **Results**, subsection “*Characteristics of Sources of Evidence*,” paragraph one:

“The first article included was published in 2007 (35), but most studies (6/8) were published between 2010 and 2019 (Table 1). The northern Indigenous communities included or mentioned were Inuit from Nunavik, Canada (3/8) (7, 11, 20), Sahtu from Northwest Territories, Canada (1/8) (34), Cree and Assiniboine from Saskatchewan, Canada (2/8) (36, 37), and unspecified Natives from Alaska, USA (2/8) (10, 35). One of the United States studies (10) also compared dog bite injuries among children from non-Nordic (American Indian) and Nordic (Alaska Native) Indigenous communities and mentioned the Navajo and other American Indian communities from the USA as well. We found no publications from Eurasia.”

A correction has been made to Table 4. The corrected table appears below.

**Table 4:** Dog bite risk factors identified by the studies included in the scoping review (some studies may have been classified in more than one categories).

	**Study design**			
**Risk factors**	**Qualitative design**	**Quantitative design**		**Reported as hypothesis or cited from literature**
	**Evidence of importance by qualitative methods**	**Statistically significant association with the occurrence of bites**	**Suggesting a possible association without having proven it**	
**Individual human factors**				
Age (children)	(11)	0	(10, 11, 34–36)	(7)
Gender (male)	0	(10), [11*]	(11, 34, 35)	(36)
Behavior toward dog (conflictual/provoked)	(7, 11, 37)	0	(11, 34, 35), [36*]	0
Sociocultural characteristics (ethnicity)	(36)	(35)	(7, 10, 11)	0
**Dog factors**				
Breeds/size	0	0	(35)	0
Function/role	(11)	0	(35)	0
Gender and reproductive status	0	0	0	(36)
Ownership or presence of a keeper	0	0	(35)	(37)
Number (lone dogs)	0	0	0	0
**Structural and environmental factors**				
Lack of veterinary service or animal control resources	(11, 36)	0	(10)	(7, 11, 34, 37)
Geographic remoteness	0	0	0	(34, 36)
Lack of legislative interventions	(7, 11)	0	(10)	(7, 36, 37)
Density of dogs in the community (overpopulation)	0	0	(10)	(7, 11, 34)
Free roaming	(7, 11), [7, 37*]	0	[35*]	(7, 37)
Seasonality (temporal variations)	(11)	(11)	(36)	0

[^*^]Contradictory result.

The publisher apologizes for this mistake. The original version of this article has been updated.

