# Supplementary material for: Occurrence and Risk Factors of Dog Bites in Northern Indigenous Communities: A Scoping Review
Source: Front Vet Sci. 2022 Apr 18;9:777640. doi: 10.3389/fvets.2022.777640 (PMC9064469; doi:10.3389/fvets.2022.777640)
Supplement: Supplementary file 1 [file Table_1.DOCX]

Supplementary Material – Appendix

**Table 1:** Complete equations used in diverse electronic databases.

| ***Themes*** | ***Dog bites*** | ***Northern communities*** |
| --- | --- | --- |
| ***Words related to the theme*** | dog bit*  dog aggression | northern  nordic  artic  autochthonous  indigenous  american indian?  native?  first nation? |
| ***Equation*** | (“northern” OR “nordic” OR “artic” OR "autochthonous" OR “indigenous” OR “american indian?” OR “native?” OR “first nation?”) AND (dog bit* OR "dog aggression") | |
